# Evaluation of a Train-The-Trainers Model for Family Peer Advocates in Children’s Mental Health

**DOI:** 10.1007/s10826-017-0961-8

**Published:** 2017-12-01

**Authors:** Kimberly Eaton Hoagwood, S. Serene Olin, Amy Storfer-Isser, Anne Kuppinger, Priscilla Shorter, Nicole M. Wang, Michele Pollock, Robin Peth-Pierce, Sarah Horwitz

**Affiliations:** 1 Department of Child and Adolescent Psychiatry, New York University Child Study Center, New York University School of Medicine, New York, NY USA; 2 Public Health Communications Consulting, LLC, Cleveland, OH USA

**Keywords:** Family peer advocates, Train-the-trainer model, Child, Adolescent, Mental health services

## Abstract

Standardized training and credentialing is increasingly important to states and healthcare systems. Workforce shortages in children's mental health can be addressed through training and credentialing of professional peer parents (called family peer advocates or FPAs), who deliver a range of services to caregivers. A theory-based training program for FPAs targeting skills and knowledge about childhood mental health services (Parent Empowerment Program, or PEP) was developed through a partnership among a statewide family-run organization, state policy leaders, and academic researchers. Prior studies by this team using highly-experienced family peer advocates (who were also co-developers of the training program) as trainers found improvements in knowledge about mental health services and self-efficacy. In 2010, to meet demands and scale the model, a training of trainers (TOT) model was developed to build a cohort of locally-trained FPAs to deliver PEP training. A pre/post design was used to evaluate the impact of TOT model on knowledge and self-efficacy among 318 FPAs across the state. Participants showed significant pre-post (6 month) changes in knowledge about mental health services and self-efficacy. There were no significant associations between any FPA demographic characteristics and their knowledge or self-efficacy scores. A theory-based training model for professional peer parents working in the children's mental health system can be taught to local FPAs, and it improves knowledge about the mental health system and self-efficacy. Studies that evaluate the effectiveness of different training modalities are critical to ensure that high-quality trainings are maintained.

## Introduction

Within the last two decades, independent reviews, as well as professional and government reports, have identified the lack of an adequately prepared mental health workforce as a key challenge to the goal of scaling evidence-based treatments (EBTs) (Hoge et al. 2009; Institute of Medicine 2006; U.S. Department of Health and Human Services, Substance Abuse and Mental Health Services Administration 2013; U.S. Public Health Service 2000; World Health Organization 2015). These reports cite a combination of worker shortages, inadequate training, limited post-professional education, and lack of quality assurance measures as factors limiting the reach of effective treatments and services.

Workforce shortages are particularly pronounced in children’s mental health (Boat et al. 2016; National Academies of Sciences, Engineering, and Medicine 2017; Stuart et al. 2009). One approach to addressing these workforce shortages is through the use of trained family support specialists, called Family Peer Advocates (FPAs) in New York state, and also known as family advocates, parent family peers, or peer support specialists nationwide. In NYS, FPA’s are parents of children with mental health needs who help other parents of children with behavioral, emotional, or mental health challenges. They provide information, support, and assist other parents in navigating the mental health care and related service systems.

Sixteen states enable family support services to be billed through Medicaid or federal block grants (Center for Health Care Strategies, Inc. 2012). The inclusion of FPAs as professionals delivering services to parents/caregivers has been found to increase the engagement of families and youth in services; increase parental knowledge about services; and increase parental expectations about quality of care (Fristad et al. 2009; Jamison et al. 2017; Kutash et al. 2011, 2013; McKay et al. 2011; Olin et al. 2010; Radigan et al. 2014).

However, integration of FPAs into the workforce also creates new challenges: ensuring that the roles, discrete functions, and competencies of the staff are clear; creating work environments and staff units that are engaged, proficient, and effective; creating clear communication channels within agencies; and creating cohesive and well-functioning teams that adequately reflect the family perspective (Hoagwood and Burns 2014). An additional challenge is that FPAs who deliver this service are employed in a variety of organizations and settings (Hoagwood et al. 2010; Walker 2008) and states are using a wide range of training requirements, with little consistency in those requirements (Center for Health Care Strategies, Inc. 2012). They range from only having lived experience to completion of formal training programs, certification, and ongoing supervision (Center for Health Care Strategies, Inc. 2012). A recent initiative by the National Federation of Families created a national-certification for Parent Support Providers (National Federation of Families 2012). These standards seek to ensure that FPAs achieve established levels of proficiency and competency, and maintain high standards of ethical and professional practice.

New York State has a 20-year history of supporting FPAs within a network of regional mental health offices located throughout the state (Hoagwood et al. 2014; Olin et al. 2010). There are approximately 200 separate family support programs, and these programs employ approximately 425 credentialed FPAs who model, coach, support, and link families to services. The family support service programs staffed with FPAs served approximately 6500 families in New York in 2015 (Kuppinger et al. 2016).

In an effort to standardize training of FPAs, the authors worked with the New York State Office of Mental Health and Families Together in NYS, a family-run organization in NYS that represents families of children with social, emotional, behavioral and cross-systems challenges, to develop definitions of core competencies for FPAs, quality indicators to assess their performance (Olin et al. 2014a), as well as a formal training and credentialing process (Kuppinger et al. 2016). The standardized training and credentialing program developed was based on a model of health activation (Green et al. 2010) and the Unified Theory of Behavior (UTB) (Jaccard et al. 1999) described fully by Olin et al. (2010). Since 2004, New York State has trained more than 780 FPAs with the model, called the Parent Empowerment Program or PEP (Hoagwood et al. 2014; Kuppinger et al. 2016; Olin et al. 2010). Details about the theory underlying the PEP model, the selection of the domains to target, and quality indicators for PEP can be found in other papers (Hoagwood and Burns 2014; Kutash et al. 2014; Olin et al. 2010, 2014a, b; Rodriguez et al. 2011).

The PEP model directly addresses barriers families face in mental health service utilization (e.g., stigma, perceptions of providers, attitudes towards mental illness, service availability, etc.) and is premised on: (1) the concept of empowerment as a process; (2) the need to engage parents in becoming active agents of change; and (3) the application of an integrated framework to empower parents, called the Parents as Agents of Change model (Olin et al. 2010). Training and consultation is co-led by a credentialed, lead peer parent trainer, and a mental health clinical provider. A key ingredient is the collaborative partnership embodied by the co-leaders, who reflect and model the respectful communication of individuals with differing perspectives, but share a common goal—namely, to improve the mental health care provided to children and families (Rodriguez et al. 2011).

The PEP training targets two broad areas: (1) skills for developing effective working relationships with families, assessing family needs, and strategies for activating families to address their children’s mental health needs; and (2) knowledge about childhood mental disorders, the diagnostic process, evidence-based treatments, and service options. The PEP training follows a manualized curriculum consisting of a core 40-h, in-person, group-based training; this curriculum is based on reviews of the literature on engagement, activation, and partnership, which was vetted through a collaborative partnership consisting of experienced FPAs, leadership of the statewide family-partnered organization, a group of mental health services researchers, and state policy leadership within New York State’s mental health authority. This structured vetting resulted in an edited book, *Improving Children’s Mental Health Through Parent Empowerment: A Guide to Assisting Parents* (Jensen and Hoagwood 2008) which lay the foundation for the curriculum. Training goals are achieved through methods based on adult learning, including direct instruction, sharing knowledge or techniques for practice, group support, modeling, vicarious learning, and practice opportunities (primarily through role rehearsals) with feedback. Core PEP training is followed by 1-h consultation calls for 6 months (12 h total) to support peer parent skill acquisition and application in their direct work with parents.

The consultation, which is co-led by the peer parent and a clinical partner (usually a licensed social worker), is structured around the Parents as Agents of Change model. Family peer advocates (FPAs) who have received the in-person training present challenging cases for consultation and feedback from trainers and their peers. The PEP conceptual framework, applying UTB activation strategies, guides the consultation (Rodriguez et al. 2011). The calls also follow the models developed for clinical consultation on evidence-based practices (Bearman et al. 2017; Gleacher et al. 2011; Nadeem et al. 2013) by including reviews of salient content (i.e., motivational interviewing principles and skills), rehearsal of specific skills (i.e., active listening), role-plays, as well as discussion of the challenging cases.

The first set of statewide PEP trainings was delivered by the PEP developers in 2006, and continued for 4 years (Olin et al. 2010; Rodriguez et al. 2011). Evaluation of a sample of 58 FPAs showed a positive impact of the training on general knowledge about mental health services and self-efficacy (Rodriguez et al. 2011). In 2010, PEP training evolved into a train-the-trainer (TOT) model that entailed close collaboration among three entities: Families Together in NYS, which took the lead in PEP trainings; Columbia University, and later New York University, to provide academic support for evaluation; and the New York State Office of Mental Health, to provide broad policy support for this work.

The TOT model followed the same principles as the original model and the same curriculum, but was launched statewide. The trainers were local FPAs who had been trained in the original model, committed to providing at least 1 year of training in their regions, willing to work closely with the state family-run organization (Families Together in NYS), and willing to work collaboratively with local clinicians (usually social workers). The TOT model entailed face-to-face trainings for 1 week, followed by 6 months of bi-weekly consultation provided by phone, which is the same intensity as the original training. Knowledge acquisition and self-efficacy were assessed prior to training, and again after the completion of the phone consultations. These domains were selected because prior evaluation of PEP indicated that knowledge about mental health services and being able to communicate that knowledge, having a sense of personal competence (self-efficacy) in understanding the mental health system, and being able to engage and work with parents were core ingredients of the program. This paper describes findings from the evaluation of the statewide PEP TOT model over a 6-year period (2010–2016).

## Method

### Participants

Families Together in NYS managed the statewide trainings. Altogether, 31 PEP trainings were held across the state: 7 in Western NY, 6 in Central NY, 4 in Upper Hudson River, 4 in Lower Hudson River, 7 in New York City, and 3 in the Long Island Region. A total of 436 individuals enrolled in these PEP trainings from 2010–2016. The average size of the 2010–2016 trainings was 19.3 (SD = 4.69).

### Procedures

While a total of 436 FPAs attended the PEP face-to-face training, this paper focuses on the 318 FPAs who attended both the face-to-face training and then completed the 6 months of bi-weekly phone consultation. All participants in this subgroup (*N* = 318) completed both the baseline and post-training questionnaires. There were no significant differences in demographic characteristics between the group who did not complete the full training (*N* = 118) and those who did complete the full training (*N* = 318). The only characteristic on which these two groups differed was primary work settings, with 31 (9.8%) of those who completed the full training reporting working primarily in school settings, and only 2 (1.7%) of those who did not complete the full training reporting working primarily in schools.

The groups were not statistically significantly different in age (45.8 ± 9.7 years for completers and 44.3 ± 9.2 years for non-completers), sex (primarily female) (95% of completers vs. 94.1% for non-completers), fluent English-speakers (98.7% of completers and 98.3% of non-completers), or experience as a family advocate (6.00 ± 6.88 years for completers and 5.36 ± 6.65 years for non-completers). We also examined potential selection bias by comparing the self-efficacy pretest scores for the completers vs. non-completers. The two-sample t-test showed no differences on the self-efficacy scores (25 original items: Δ = 0.06, SD = 0.38, *p* = .12; 10 new items: Δ = 0.04, SD = 0.46, *p* < .40) or the general knowledge scores (Δ = 0.5, SD = 4.1, *p* = .29) (results not shown).

### Measures

FPAs who participated in the PEP training were assessed before the in-person training began (baseline), and after the 6 months of consultation calls were completed (post-training). Assessments were collected from all FPAs, regardless of whether or not they completed the entire PEP training. Participants completed questionnaires consisting of three measures, which collected information on demographics, PEP training knowledge, and self-efficacy.

The first measure was a 28-item demographic questionnaire to collect data on advocates’ backgrounds and life experience, and work and work settings (completed at baseline only). The second measure was a general knowledge test to measure objective knowledge of content. These questions were keyed to the content within the six PEP training modules and consisted of 34 multiple choice, true/false, and fill-in-the-blank questions (knowledge is calculated as the percent of correct responses, ranging from 0 to 1, with 1 meaning 100% correct).

The third measure was the Vanderbilt Mental Health Services Efficacy (VMHSE) Scale (Bickman et al. 1991), modified to measure the degree to which FPAs feel effective in helping parents access child mental health services. Twenty-five items are scored on a 5-point scale, ranging from 1 (strongly disagree) to 5 (strongly agree). Typical items include, “I believe that I can help parents access and use effectively mental health services for their children” and “My skills in dealing with mental health services will help me to empower parents to change things that might be wrong with their child’s treatment”. Mental health self-efficacy is the mean score of summed items. Alpha reliability for the VMHSE was .89. Ten additional items related to partnering with parents were developed and added to this scale specifically for this study. The total score for the 25-item and 10-item scales were calculated for participants who answered at least 75% of the items. Higher scores indicate greater self-efficacy.

### Data Analysis

The data were summarized using means and standard deviations for normally distributed measures, median and interquartile range for skewed measures, and counts and percentages for categorical measures. The two-sample *t*-test and chi-square test were used to compare demographic characteristics of PEP participants who did and did not complete PEP training. Paired t-tests and McNemar’s test were used to compare pretest and posttest scores for those who completed PEP training. Statistical significance was set at *p* = .05 and no adjustments were made for multiple comparisons. SAS version 9.4 was used for these analyses (SAS Institute Inc. 2011).

## Results

Of the 318 participants who completed the entire training, 98.3% spoke English fluently, 14.5% spoke Spanish, and 16% spoke another language. The majority of this group (29.2%) had attained an associate’s degree, other 2-year degree, or some college, followed by similar proportions who did not complete high school, had a high school diploma or a GED (29.7%), and those who had a Bachelor’s degree (26%). Of this group, 82.4% had navigated the education or special education system on behalf of their child or family, 79.9% had navigated the mental health system, and 63.8% had navigated the health system. The sample was 45.8 years old on average; two-thirds were Caucasian, and nearly all were female (95%) (see Table 1).

**Table 1:** Characteristics of individuals who completed PEP training

	(*n* = 318)
Age	45.8 ± 9.7
Female	301 (95.0)
Race/ethnicity	
Caucasian	215 (67.6)
Latino/Hispanic	47 (14.8)
African American	39 (12.3)
Other	17 (5.3)
Languages spoken fluently	
English	314 (98.7)
Spanish	46 (14.5)
Other language (not English or Spanish)	16 (5.0)
Education	
Did not complete high school/High school diploma/GED	85 (26.7)
Associates degree/2 year degree/some college	93 (29.2)
Bachelor’s degree	82 (26.0)
Some graduate school	16 (5.1)
Graduate degree/professional degree	39 (12.4)
Years’ experience as a family advocate	6.00 ± 6.88
Working as a paid family advocate	
Paid full-time	183 (58.1)
Paid part-time	107 (34.0)
Unpaid	25 (7.9)

General knowledge scores were significantly higher after PEP training compared to baseline (i.e., before PEP training) (Δ = 1.3, SD = 3.4, *p* < .001). Examination of the individual items showed significant gains on parents as agents of change knowledge questions (e.g., the Parents as Agents of Change model advocates problem-solving as a first step to working with parents, true or false). Self-efficacy scores were also significantly higher after completing PEP training compared to scores prior to PEP training for both the 25 original items (Δ = 0.12, SD = 0.38, *p* < .001) and for the 10 new items (Δ = 0.08, SD = 0.51, *p* < .01) (Table 2). For the 25 original items, the greatest improvements in self-efficacy were on helping parents get mental health services (e.g., I have made an important difference in helping parents get the mental health services their child has received). For the 10 new items, the greatest gains were related to building partnerships (e.g., I can coach families to partner with professionals). There were no significant associations between any FPA demographic characteristics (e.g., age, education, region of state, work setting or type of supervisor) and their performance on general knowledge or self-efficacy scores.

**Table 2:** PEP general knowledge and self-efficacy results

	*N*	Pretest score	Posttest score	Difference	*p*-value
General knowledge scores					
Number correct out of 34 items	315	26.1 ± 4.0	27.4 ± 3.7	1.3 ± 3.4	**<.001**
Self-efficacy scores					
25 original items	308	4.28 ± 0.37	4.40 ± 0.35	0.12 ± 0.38	**<.001**
10 new items	302	4.30 ± 0.45	4.38 ± 0.47	0.08 ± 0.51	**.006**

*Note*: Mean ± SD shown. Scores calculated if ≥75% of the items were completed

Bold values are statistically significant at the *p* < .05 level

## Discussion

As states move towards managing services using value-based payment approaches (Centers for Medicare and Medicaid Services 2017) and mandating workforce competencies (Boat et al. 2016; National Academies of Sciences, Engineering, and Medicine 2017; Stuart et al. 2009), feasible and generalizable training for all service providers will become increasingly essential. FPAs are an important group of providers whose work improves family engagement, access, and knowledge about quality (Fristad et al. 2009; Jamison et al. 2017; Kutash et al. 2013; Kutash et al. 2011; McKay et al. 2011; Olin et al. 2010; Radigan et al. 2014). The use of a train-the-trainer (TOT) model is one way to make training of this important workforce more affordable and feasible.

This evaluation of a TOT model replicated the findings of the original PEP training study (Rodriguez et al. 2011), which involved training by individuals involved in the development of the original PEP training model, with an experienced family parent trainer and a licensed social work trainer. The TOT model was delivered by trainers who were local FPAs and local clinicians who had been trained in the original model, and had committed to providing at least one training during the following year to new FPAs in their region. The original PEP training model involved 58 PEP participants who received standardized PEP training, while the current evaluation was based on a TOT approach that included a much larger sample of 318 FPAs. Across the evaluation of both training models, PEP participants showed significant pre-post (6 month) changes in knowledge and self-efficacy.

The replication of the evaluation findings of these two training models is important because the impact of TOT models is often weaker than findings from models using highly-trained and often academically-based trainers. The fact that the statewide evaluation found a positive effect on both knowledge gains and self-efficacy, replicating the original PEP training study, suggests that a TOT approach for improving knowledge, skills, and competencies of FPAs across a state with wide regional variation has promise as a feasible and scalable model.

### Limitations

As with any pre-post evaluation that lacks a control or comparison group, this study’s findings must be interpreted with caution. The changes noted might be due to sample bias, historical changes that would occur irrespective of the training, and non-program influences on outcomes. Furthermore, the use of self-report measures substantially weakens the findings because participants sometimes overestimate their knowledge, attitudes, or skills. Nevertheless, these findings are based on a much larger sample, and are consistent with the earlier evaluation of trainings conducted by the original trainers.

The shortage of mental health clinical specialists, combined with increasing rates of behavioral health needs among children and families, creates the conditions for a health crisis. Standardized training and credentialing, particularly among the paraprofessional workforce, is increasingly important as states and healthcare systems move towards adoption of credentialing standards to improve the quality of services. Strengthening the peer parent workforce through theory-based and competency-based training to work effectively across community and hospital-based services is important for enhancing the quality, robustness, and viability of the children’s mental health system.

A theory-based training model for improving workforce competencies among professional peer parents in the children’s mental health system is thus an important step forward in addressing this gap. As training models evolve, efforts to demonstrate the replicability of training effectiveness will continue to be important in the future. In the last two decades, training models have evolved from tightly-controlled trainings, often delivered in-person by individuals closely tied to the training developer, to TOT models and, increasingly, the use of virtual trainings. As newer web-based training models are developed to meet increased demands for lower costs and accessibility, studies that evaluate the effectiveness of different training modalities are critical to ensure that high-quality trainings are maintained.
